# Circ-ATL1 silencing reverses the activation effects of SIRT5 on smooth muscle cellular proliferation, migration and contractility in intracranial aneurysm by adsorbing miR-455

**DOI:** 10.1186/s12860-022-00461-2

**Published:** 2023-01-30

**Authors:** Jichong Xu, Chun Fang

**Affiliations:** Department of Interventional Radiology, Tongji Hospital of Tongji University, 389, Xincun Road, Shanghai, 200065 China

**Keywords:** Circ-ATL1, miR-455, SIRT5, Intracranial aneurysm, Smooth muscle cells

## Abstract

**Background:**

Alterations in vascular smooth muscle cells (VSMCs) contribute to the pathogenesis of intracranial aneurysms (IAs). However, molecular mechanisms underlying these changes remain unknown. The present study aimed to characterize the molecular mechanisms underlying VSMC-mediated IAs.

**Methods:**

Expression of the circular RNA circ-ATL1 and microRNA miR-455 was detected in IAs by RT-qPCR. Interactions between circ-ATL1, miR-455 and SIRT5 were examined by luciferase reporter analysis and RT-qPCR. The regulatory roles of circ-ATL1, miR-455 and SIRT5 in VSMC migration, proliferation and phenotypic modulation were also examined by CCK8, Transwell® migration and western blot assays.

**Results:**

Biochemical and bioinformatic techniques were used to demonstrate that circ-ATL1 and miR-455 participated in disparate biological processes relevant to aneurysm formation. Clinically, increased expression of circ-ATL1 and downregulated miR-455 expression were observed in IA patients compared with healthy subjects. Silencing of circ-ATL1 led to suppression of VSMC migration, proliferation and phenotypic modulation. Both SIRT5 and miR-455 were found to be downstream targets of circ-ATL1. SIRT5 upregulation or miR-455 inhibition reversed the inhibitory effects induced by circ-ATL1 silencing on VSMC proliferation, migration and phenotypic modulation. We found that VSMC phenotypic modulation by circ-ATL1 upregulation and miR-455 downregulation had a critical role in the development and formation of AIs. Specifically, circ-ATL1 downregulation reversed IA formation.

**Conclusion:**

Our data provide the theoretical basis for future studies on potential clinical treatment and prevention of IAs.

**Supplementary Information:**

The online version contains supplementary material available at 10.1186/s12860-022-00461-2.

## Background

Unruptured intracranial aneurysms (UIAs) are relatively common in the general population, occurring in ~ 3.2% (95% confidence interval [CI], 1.9–5.2%) of the adult population (mean_age_ = 50) worldwide. Due to widespread use of high-resolution magnetic resonance imaging (MRI), the incidental detection of UIAs is becoming more frequent. The majority of UIAs will not rupture. For example, out of 1 million adults in the population with a mean age of 50, around 32,000 harbor UIAs, and only 0.25% of these, i.e., 1 per 200 to 400 will rupture [1–3]. No treatment guidelines for UIAs have been tested clinically. Case reporting is crucial to better understand the potential ethnic predispositions and underlying pathophysiology of the disease and allow for improved patient selection for intervention [4]. Although previous studies have reported that phenotypic alternations in vascular smooth muscle cells (VSMCs) including proliferation are involved in IA pathogenesis [5], the molecular mechanisms underlying VSMC phenotypic modulation remain unknown.

Circular RNAs (circRNAs) have a significant role in mediating IA progression [6]. circRNAs belong to a family of non-coding RNA (ncRNA) molecules that contain a continuous covalently closed loop that protects against RNA exonuclease degradation [7–9]. circRNAs originate from introns, intergenic regions in eukaryotes or exons, which exhibit tissue-specific expression patterns [10, 11]. Previously, circ_0020397 has been shown to regulate VSMC viability through up-regulation of GREM1 by miR-502-5p in IA [12], while hsa_circ_0021001 levels in peripheral blood may be a potential marker in IA screening [13]. circ-ARFIP2 regulates migration, invasion and proliferation of human VSMCs through miR-338-3p-dependent KDR modulation [14]. Hsa_circ_0102049 (also named circ-ATL1) has also been reported to have a critical role in mediating pancreatic ductal adenocarcinoma progression via targeting miR-455 [15]. In addition, multiple studies have reported that dysregulated microRNAs (miRNAs) may have a pathogenic role in IAs. Disruption of the protein translation process may also have a pathogenic role in the development of IAs [16]. Previously, miR-455 has been shown to inhibit cell proliferation [17]. However, the role of hsa_circ_0102049/miR-455 in IA progression remains unknown.

The present study aimed to characterize the roles of hsa_circ_0102049 and miR-455 in regulating smooth muscle cell migration, proliferation and phenotypic modulation. Our findings provide a theoretical basis for future studies on the potential clinical treatment and prevention of IA.

## Methods

### Ethics statement

Thirty IA patients from the Interventional Neuroradiology Department of Tongji Hospital at Tongji University (Shanghai, China) were recruited into this study between June 2019 and March 2021. Aneurysm sizes ranged from 2.5 × 1.5 mm to 20 × 15 mm. Age-matched healthy subjects without a family history of IA were included as controls (*n* = 30). No significant differences were found between the two groups in terms of aneurysm size and patient age (*P* > 0.05). Blood samples were collected from all subjects. Samples were centrifuged at 2800×*g* for 5 min after standing for 0.5 h at room temperature (RT). Serum samples were divided into aliquots, which we maintained at − 80 °C. IA tissues were removed during clipping surgery, and snap-frozen in liquid N_2_. Normal superficial temporal arteries were collected from trauma patients receiving craniotomy treatments.

### Overexpression and interference vector constructs

Human siRNA against circ-ATL1 (si-circ-ATL1), the miR-455 inhibitor (miR-455 inhibitor: 5′–GUGUAUAUGCCCAUGGACUGC–3′) and negative control inhibitor (inhibitor-NC: ACGUGACACGUUCGGAGAATT) were obtained from GenePharma (Shanghai, China). For SIRT5 overexpression, we constructed a SIRT5 eukaryotic expression vector (SIRT5/pcDNA3.1) by placing the SIRT5 open reading frame into pcDNA3.1(+). For luciferase reporters, SIRT5 3′-UTR and circ-ATL1 were amplified from human genomic DNA. We mutated miR-455 binding sites within *SIRT5* 3′-UTR and circ-ATL1 to eliminate complementarity. Mutant (MUT) or wild-type (WT) *SIRT5* 3′-UTR and circ-ATL1 were cloned into psiCHECK-2 luciferase vectors for the luciferase reporter assays. The plasmids were confirmed through sequencing.

### Cell culture and transfection

Human VSMCs (ATCC, Manassas, VA, USA) were cultured in Dulbecco’s Modified Eagle Medium (DMEM) containing 10% fetal bovine serum (FBS), glutamine and penicillin/streptomycin at 37 °C in an humidified atmosphere of 5% CO_2_. VSMCs were transfected with miR-455 inhibitor or si-circ-ATL1 at a concentration of 50 nM using Lipofectamine 2000 (Invitrogen, Carlsbad, CA, USA) according to the manufacturer’s protocol. For SIRT5 overexpression, VSMCs were transfected with 1 μg SIRT5 plasmid or control vector. Transfected cells were used in subsequent experiments 2 days after transfection.

### Subcellular fractionation

Nuclear-cytoplasmic RNA fractionation was performed using a Nuclear and Cytoplasmic Extraction Reagents Kit (Thermo Fisher Scientific) following the manufacturer’s protocol. We extracted and captured circ-ATL1 and linear ATL1 in nuclear and cytoplasmic VSMC fractions. U6 or GAPDH were used as internal controls.

### Bioinformatic analyses

Circular RNA Interactome was used to identify circRNA and miRNA target genes. TargetScan was utilized to predict interactions between miRNAs.

### Fluorescence in situ hybridization (FISH)

Geneseed Biotech (Guangzhou, China) constructed specific probes to circ-ATL1 (Dig-5′-GGAAAATTGAAAGAGTCTGATATTTCTTG-3′-Dig). Signals were captured through FITC-conjugated anti-digoxin anti-biotin antibodies (Jackson ImmunoResearch Inc., West Grove, PA, USA). Nuclei were counterstained using 4,6-diamidino-2-phenylindole (DAPI). Figures were plotted using a Zeiss LSM 700 confocal microscope (Carl Zeiss, Oberkochen, Germany).

### Western blot analysis

Protein was extracted from cells or tissues using lysis buffer containing protease inhibitors. Samples were centrifuged at 12,000×*g* at 4 °C. Protein concentrations were determined using a BCA Kit (Pierce, Rockford, IL, USA). Protein samples (40 μg) were separated by SDS-PAGE, then transferred to polyvinylidene difluoride membranes. After blocking in 5% fat-free milk, membranes were incubated overnight at 4 °C with the following primary antibodies: anti-MMP-3, anti-SM-MHC, anti-SM-α-actin, anti-MMP-2, anti-SM22 and anti-GAPDH. The antibodies were purchased from Santa Cruz Biotechnology (Santa Cruz, CA, USA), and used at dilution of 1:1000. Membranes were incubated for 1 h at RT with horseradish peroxidase-conjugated secondary antibodies (Santa Cruz Biotechnology). Samples were visualized using an Enhanced Chemiluminescence Detection Kit (Amersham Biosciences, Piscataway, NJ, USA). Protein bands were normalized against loading controls, and quantified using the Quantity One program (Bio-Rad, Hercules, CA, USA).

### Cell proliferation assay

To examine the effects of circ-ATL1, miR-455, and SIRT5 on VSMC proliferation, we transfected VSMCs with circ-ATL1, miR-455, and SIRT5 separately or in combination. Cells were seeded onto six 96-well plates at a density of 2 × 10^3^ cells/well. Proliferation levels were detected using a CCK8 kit (Dojindo Laboratories, Kumamoto, Japan) and enzyme-linked immunosorbent assay reader (Thermo Labsystems, MA, USA) following the manufacturer’s instructions. Data were collected from > = 3 independent experiments that had been carried out in triplicate.

### Cell migration assay

Cells that had been transfected in 200 μL serum-free DMEM medium were resuspended and seeded onto Boyden chambers (Corning, Corning, NY, USA) containing a 8.0-μm pore membrane (5 × 10^4^ cells/well) for migration assays. Cells were incubated in DMEM containing 20% FBS at 37 °C in an atmosphere of 5% CO_2_. After 1 day, cells adhering to the lower chamber surface were fixed, while cells on the upper surface were removed. After staining with 0.05% Crystal Violet solution, cells were counted in three randomly selected high-power fields by light microscopy (Olympus, Tokyo, Japan). Migration assays were carried out in triplicate.

### RNA extraction and RT-PCR assays

Total RNA was extracted from IA serum or VSMCs using TRIzol reagent (Invitrogen). Reverse transcription was carried out using the Superscript First Strand cDNA Synthesis System (Invitrogen). We performed RT-PCR using the One Step SYBR® Prime Script TM RT-PCR Kit II (Takara, Tokyo, Japan). GAPDH and U6 were used as the internal controls in mRNA and miRNA assays. Results were analyzed using the 2^−ΔΔCt^ method. The following primers were used: GAPDH (forward primer: 5′-AAGCTCACTGGCATGGCCTT-3′; reverse primer: 5′-CGGCATGTCAGATCCACAAC-3′); U6 (forward primer: 5′-CTCGCTTCGGCAGCACA-3′; reverse primer: 5′-AACGCTTCACGAATTTGCGT-3′); circ-ATL1 (forward primer: 5′-GATGGAAAATTGAAAG-3′; reverse primer: 5′-CCAGTCTCGAACAAG-3′); miR-455 (forward primer: 5′-ACACTCCAGCTGGGGCAGTCCACGGGCATATACAC-3′; reverse primer: 5′-GTGCAGGGTCCGAGGT-3′); SIRT5 (forward primer: 5′-GTCATCACCCAGAACATCGA-3′; reverse primer: 5′-ACGTGAGGTCGCAGCAGCAAGCC-3′).

### Luciferase assay

miRNA target validation assays were carried out as described previously [15]. Briefly, we seeded VSMCs into 12-well plates at a density of 120,000 cells/well, 1 day before transfection. Cells were co-transfected with 100 ng empty psiCHECK-2 vector or psiCHECK-2 + miR-455, or control vector constructs using Lipofectamine 2000 following the manufacturer’s protocol. We constitutively utilized expressed the firefly luciferase gene in the psiCHECK-2 vector as the normalization control. Three days after transfection, *Renilla* and firefly luciferase activities were measured concomitantly with a luminometer (Promega, Madison, WI, USA).

### Statistical analyses

Results are expressed as mean ± standard mean error (SME). We employed the two-tailed Student’s *t*-test to compare differences among groups with GraphPad Prism (GraphPad Software, La Jolla, CA, USA). *P* < 0.05 was considered to be statistically significant.

## Results

### Circ-ATL1 and miR-455 are involved in IA progression

**Fig. 1:**
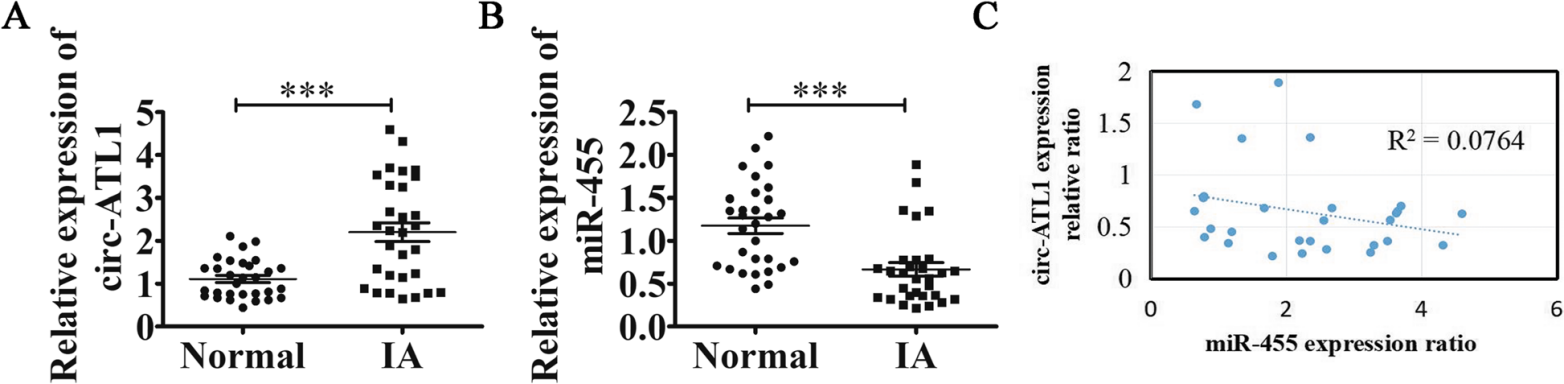
Expression of circ-ATL1 and miR-455 in healthy and IA patients. **A** and **B** Levels of circ-ATL1 (**A**) and miR-455 (**B**) in the serum were assessed by RT-qPCR. Results are presented as mean ± SD. ^***^P < 0.001 healthy vs. normal controls. *n* = 30. **C** Pearson correlation analysis was performed to determine the correlation between circ-ATL1 and miR-455 expression (R^2^ = 0.0764, *n* = 30)

circ-ATL1 expression was found to be significantly increased (2-fold increase, *P* < 0.001) in IA patients compared with healthy controls (Fig. 1A), while miR-455 expression was significantly lower in IA patients (2-fold decrease, *P* < 0.001) compared to healthy controls (Fig. 1B). The correlation between circ-ATL1 and miR-455 expression was examined using the Pearson correlation test. As shown in Fig. 1C, a negative correlation (R^2^ = 0.0764, *P* < 0.05) was observed between circ-ATL1 and miR-455 expression. Taken together, our data suggest that miR-455 and circ-ATL1 have a role in IA progression.

### Silencing circ-ATL1 expression suppresses VSMC migration and proliferation

**Fig. 2:**
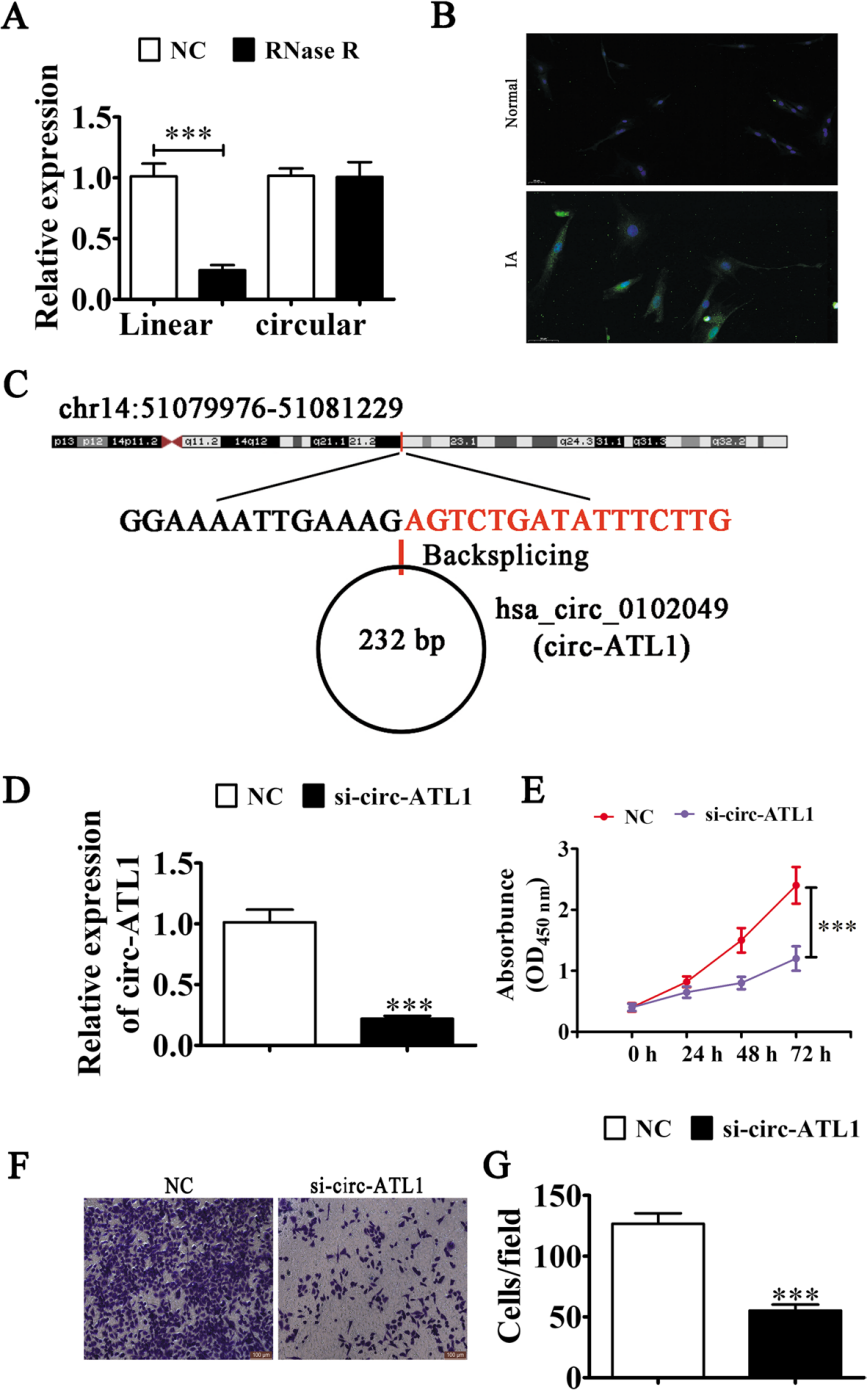
The effects of circ-ATL1 on VSMC viability. **A** RT-qPCR analysis of circ-ATL1 and linear ATL1 expression after RNase R treatment in VSMCs. Data are presented as mean ± SD. ^***^P < 0.001 vs NC. *n* = 3. **B** FISH analysis was used to show the expression and subcellular localization of circ-ATL1 in VSMCs both healthy and IA patients. **C** *ATL1* gene and circ-ATL1 genomic loci. **D** RT-qPCR analysis of circ-ATL1 expression in VSMCs transfected with siRNA against circ-ATL1 (si-circ-ATL1). Results are presented as mean ± SD. ^***^P < 0.001 versus NC. *n* = 3. **E** The effects of circ-ATL1 on VSMC proliferation were assessed using the CCK8 assay. Results are presented as mean ± SD. ^***^P < 0.001 versus NC. *n* = 3. (**F** and **G**) The effects of circ-ATL1 on VSMC migration were examined using the Transwell® migration assay. Results are presented as mean ± SD. ^***^P < 0.001 versus NC. *n* = 6

Since circ-ATL1 was found to be upregulated in IA patients, we hypothesized that circ-ATL1 might have a critical role in regulating the function of VSMCs in IA patients. We found that circ-ATL1 in proliferating VSMCs was resistant to degradation through RNase R (5-fold decrease, *P* < 0.001), although the linear transcript was significantly digested by RNase R (Fig. 2A). FISH analysis revealed that circ-ATL1 expression was upregulated in IA patients and predominantly located in the cytoplasm (Fig. 2B). Bioinformatics analysis found that circ-ATL1 (hsa_circ_0102049) was located in chr14:51079976–51,081,229, 232 bp from the origin of the ATL1 gene exon. Thus, hsa_circ_0102049 is also known as circ-ATL1 (Fig. 2C). To characterize the role of circ-ATL1 in VSMC function, we transfected VSMCs with siRNA against circ-ATL1 (si-circ-ATL1) or negative control (NC). We found that circ-ATL1 expression was downregulated (5-fold decrease, *P* < 0.001) after si-circ-ATL1 treatment compared to control cells (Fig. 2D). The CCK8 assay showed that silencing circ-ATL1 expression inhibited VSMC proliferation (2-fold decrease, *P* < 0.001) compared to control cells (Fig. 2E). Furthermore, downregulation of circ-ATL1 significantly suppressed migration of VSMCs (2-fold decrease, *P* < 0.001) in the Transwell® migration assay (Fig. 2F, G).

### The role of circ-ATL1 in VSMC phenotypic modulation

**Fig. 3:**
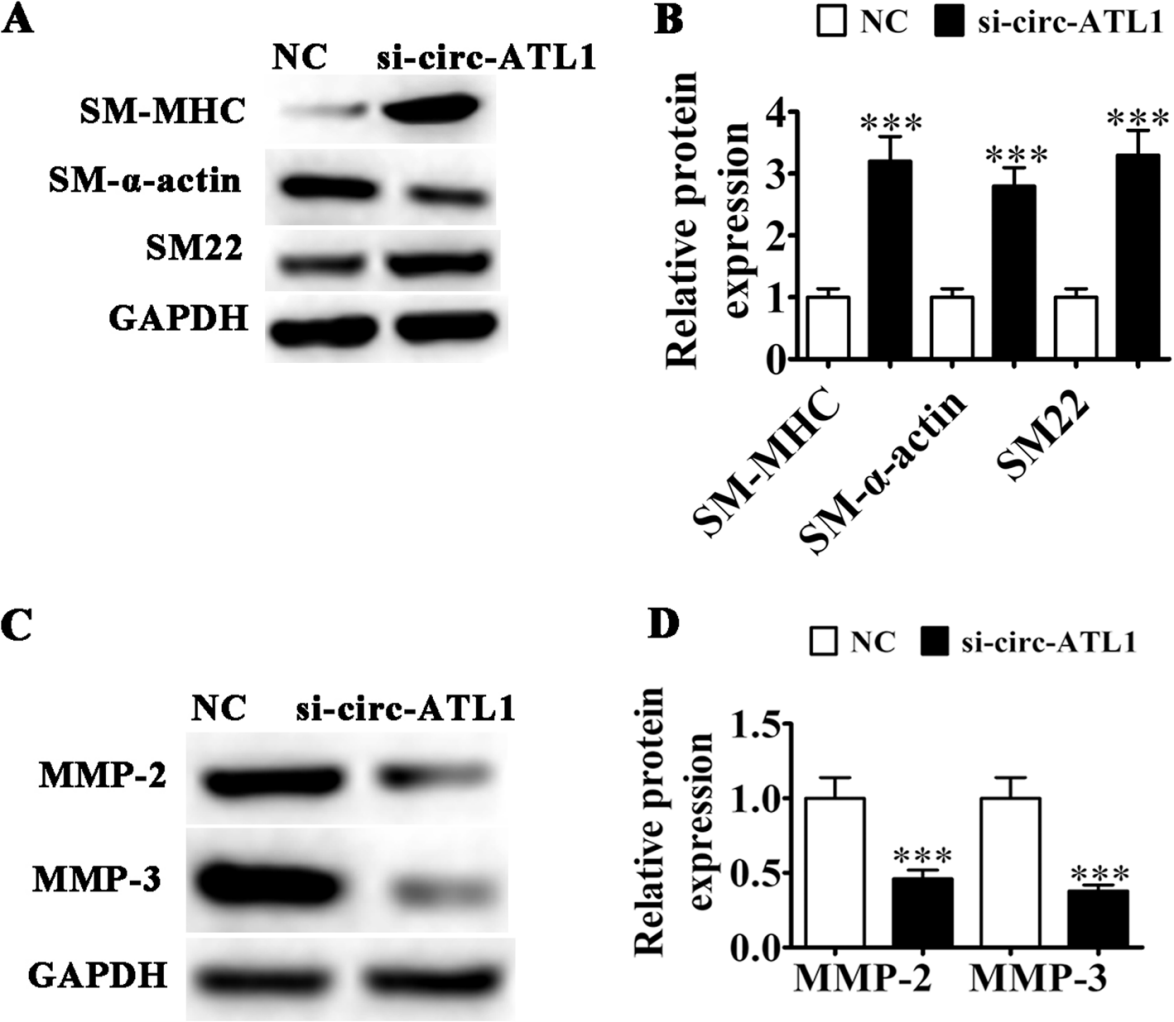
The role of circ-ATL1 in VSMC phenotypic modulation. **A** Western blot analysis of contractile protein expression (SM-MHC, SM22 and SM-α actin) in VSMCs after si-circ-ATL1 transfection. *n* = 3. **B** Quantification of relative protein expression. Results are presented as mean ± SD. ***P < 0.001 versus NC. **C** WB analysis showing MMP-3 and MMP-2 expression in VSMCs after si-circ-ATL1 transfection. *n* = 3. **D** Quantification of relative protein expression levels. Results are presented as mean ± SD. ***P < 0.001 versus NC

We next sought to determine the role of circ-ATL1 in phenotypic modulation in cultured VSMCs. We found that circ-ATL1 downregulation caused a significant increase in the expression of contractile proteins such as smooth muscle myosin heavy chain (SM-MHC), SM22 and SM-α-actin (3-fold increase, *P* < 0.001) (Fig. 3A and B), while a decrease in matrix metalloproteinase-3 (MMP-3) and MMP-2 expression (2-fold decrease, *P* < 0.001) was observed (Fig. 3C and D).

### Both SIRT5 and miR-455 are targets of circ-ATL1

**Fig. 4:**
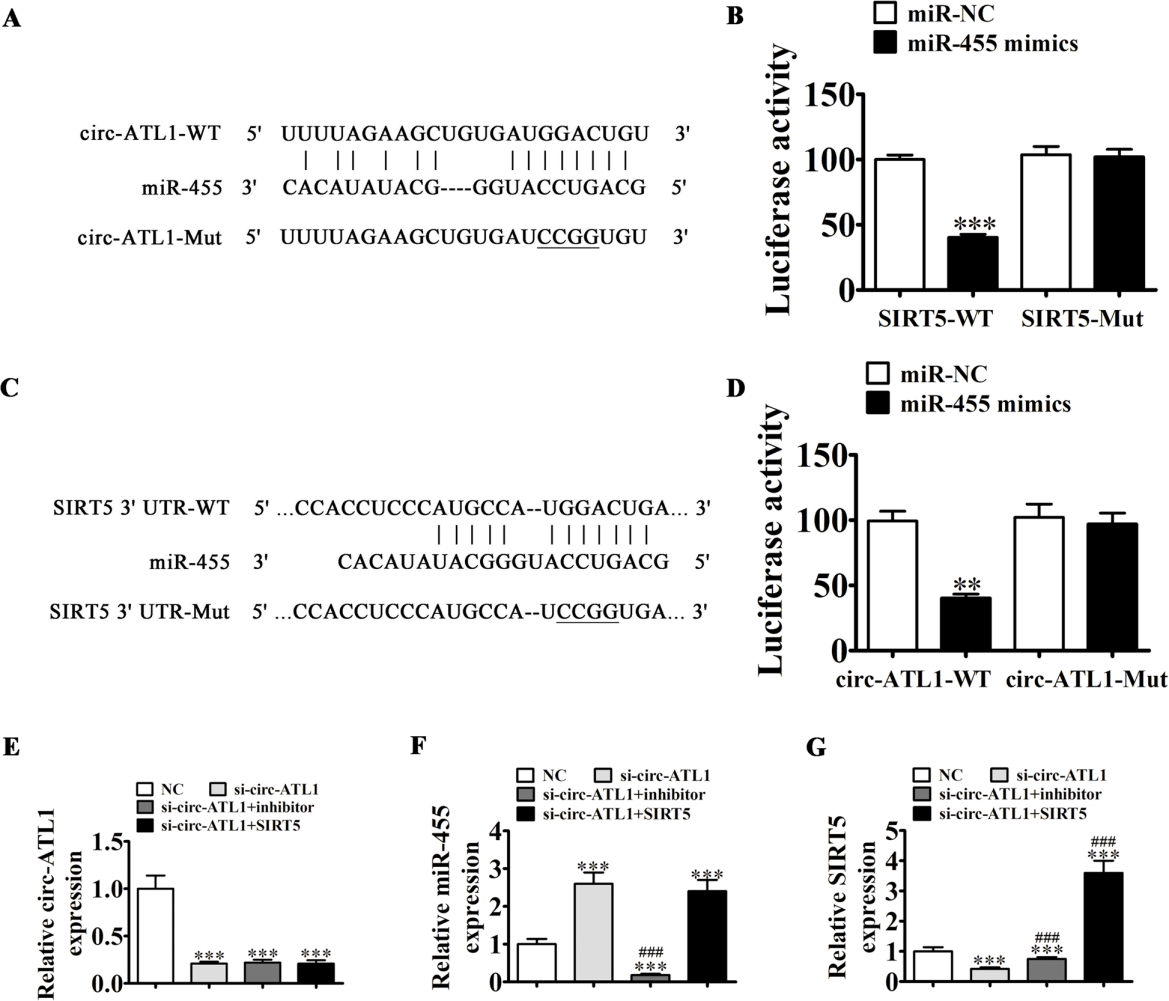
Both SIRT5 and miR-455 are targets of circ-ATL1. **A** BioEdit software was employed to identify potential miR-455 binding sites within circ-ATL1. The MUT circ-ATL1 form is shown. **B** Relative luciferase activity was detected 2 days after VSMCs were transfected with miR-455 mimic/normal control (NC) or circ-Astn1 WT/MUT. Results are presented as mean ± SD. ^***^P < 0.01. *n* = 3. **C** Prediction of miR-455 binding sites within SIRT5 3′-UTR. The 3′-UTR-SIRT5 MUT form is shown. **D** Relative luciferase activity was detected 2 days after VSMCs were transfected with miR-455 mimic/NC or 3′-UTR-SIRT5 WT/MUT. Results are denoted by mean ± SD. **P < 0.01. *n* = 3. **E-G** circ-ATL1 (**E**), miR-455 (**F**), and SIRT51 (**G**) expression levels were measured by RT-qPCR. Results are presented as mean ± SD. ^***^P < 0.001 vs. NC. ^###^*P* < 0.001 vs. si-circ-ATL1. *n* = 3

Since our bioinformatics analysis revealed that circ-ATL1 interacts with miR-455, we next examined whether miR-455 was a target of circ-ATL1 using a luciferase reporter vector that contained the circ-ATL1 sequence in VSMCs transfected with miR-455 mimics (Fig. 4A). We found that miR-455 inhibited the luciferase activity in circ-ATL1 WT cells (2-fold decrease, *P* < 0.001), but not in circ-ATL1 MUT cells (Fig. 4B), suggesting that miR-455 was a target of circ-ATL1.

Bioinformatics analysis also inferred that SIRT5 was a downstream target of miR-455. To further understand the correlation between miR-455 and SIRT5, we cloned MUT or WT 3′-UTR-SIRT5 sequences containing the miR-455 binding sequence into a luciferase reporter vector (Fig. 4C). VSMCs were transfected with the luciferase reporter vector in the presence or absence of the miR-455 mimic. Our luciferase reporter results revealed that miR-455 inhibited luciferase activity in 3′-UTR-SIRT5 WT cells (2-fold decrease, *P* < 0.001), but not in 3′-UTR-SIRT5 MUT cells (Fig. 4D), suggesting that SIRT5 was a target of miR-455 target.

Treatment with si-circ-ATL1 successfully suppressed circ-ATL1 expression (3-fold decrease, *P* < 0.001) as measured by RT-qPCR. Furthermore, treatment with the miR-455 inhibitor or SIRT5 overexpression vector did not affect circ-ATL1 expression in VSMCs (Fig. 4E), suggesting that miR-455 and SIRT5 were downstream of circ-ATL1. RT-qPCR demonstrated that circ-ATL1 knockdown increased miR-455 expression (2.5-fold increase, *P* < 0.001). However, overexpression of SIRT5 did not affect circ-ATL1-induced miR-455 expression (Fig. 4F), suggesting that miR-455 was downstream of circ-ATL1. We also found that circ-ATL1 silencing led to a decrease in SIRT5 expression (2-fold decrease, *P* < 0.01). Downregulation of miR-455 reversed this circ-ATL1-induced inhibitory effect on SIRT5 expression. Transfection with the SIRT5 overexpression vector led to a significant increase in SIRT5 expression (Fig. 4G). Taken together, our data demonstrate that circ-ATL1 enhances SIRT5 expression by sponging miR-455.

### Upregulation of SIRT5 or inhibition of miR-455 restores the migratory, proliferative and phenotypic modulation abilities of VSMCs after circ-ATL1 silencing

**Fig. 5:**
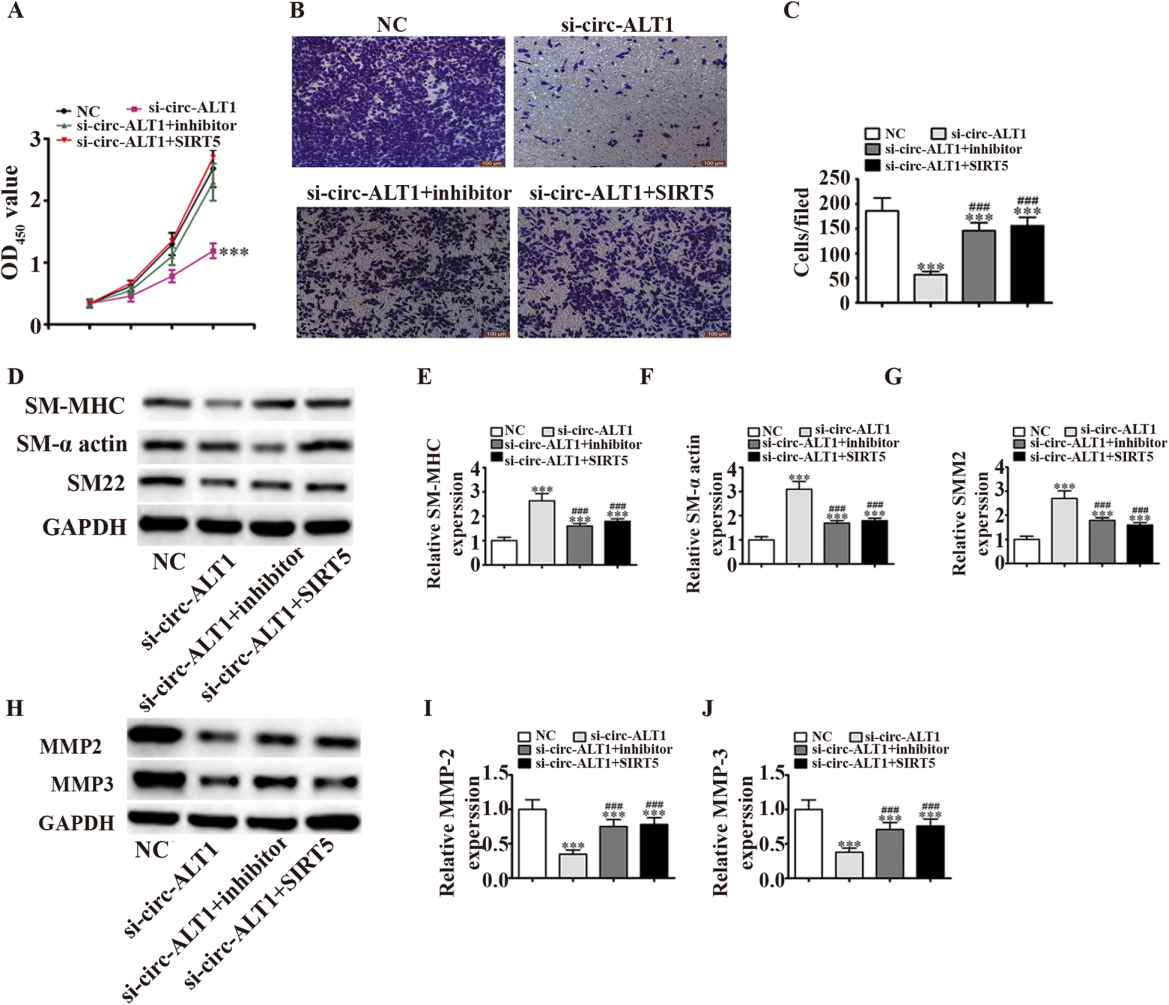
Upregulation of SIRT5 or miR-455 inhibition reverses the inhibitory effects of circ-ATL1 silencing on VSMC migration and proliferation. **A** The effects of circ-ATL1 on VSMC proliferation were detected using the CCK8 assay. Results are presented as mean ± SD. ^***^P < 0.001 versus NC. *n* = 3. **B** and **C** The effects of circ-ATL1 on VSMC migration were determined using the Transwell® migration assay. Results are presented as mean ± SD. ^***^P < 0.001 versus NC. ^###^*P* < 0.001 vs. si-circ-ATL1. *n* = 3. **D** Contractile protein (SM-MHC, SM-α actin, and SM22) expression levels were measured in VSMCs transfected with si-circ-ATL1 by WB analysis. *n* = 3. **E–G** Quantification of relative protein expression levels. Results are presented as mean ± SD. ***P < 0.001 versus NC. ^###^*P* < 0.001 vs. si-circ-ATL1. **H** WB analysis of MMP-3 and MMP-2 expression levels in VSMCs transfected with si-circ-ATL1. *n* = 3. **H** and **J** Quantification of relative protein expression levels. Results are presented as mean ± SD. ***P < 0.001 versus NC. ^###^*P* < 0.001 vs. si-circ-ATL1

Since circ-ATL1 was upregulated in IA patients, we hypothesized that circ-ATL1 might have a critical role in regulating VSMCs. To determine the role of circ-ATL1 in regulating VSMC functions, we transfected VSMCs with either a negative control (NC) or the circ-ATL1 silencing vector to decrease circ-ATL1 expression. The CCK8 assay revealed that downregulation of circ-ATL1 inhibited VSMC proliferation (2-fold decrease, *P* < 0.001), whereas upregulation of SIRT5 or inhibition of miR-455 reversed this inhibitory effect (Fig. 5A). The Transwell® migration assay demonstrated that SIRT5 upregulation or inhibition of miR-455 restored the VSMC migration ability after circ-ATL1 silencing (Fig. 5B and C). Next, the potential effects of circ-ATL1, miR-455 and SIRT5 on phenotypic modulation were examined in cultured VSMCs. Western blot analyses demonstrated that SIRT5 upregulation or miR-455 inhibition reversed the activation effects of circ-ATL1 silencing on the expression of contractile proteins, including SM-MHC, SM22 and SM-α-actin (Fig. 5D–G). In addition, SIRT5 upregulation or miR-455 inhibition reversed the inhibitory effects of circ-ATL1 silencing on MMP-3 and MMP-2 expression (Fig. 5H–J).

## Discussion

Saccular UIAs have a prevalence of 3% in the adult population, and have been diagnosed at a higher frequency due to improved cranial imaging quality [2]. Although many studies have focused on aneurysm progression, rupture and development, less is known about the efficacy and risks of preventive treatment [18, 19]. Previous studies have found that circ-ATL1 expression promoted the progression of pancreatic ductal adenocarcinoma by functioning as a miR-455 sponge [15]. Here, we found that circ-ATL1 expression was increased in IA patients, while miR-455 expression was decreased. We also demonstrated that downregulation of circ-ATL1 inhibited the migration, proliferation and phenotypic modulation of VSMCs. Our findings are consistent with previous studies, which reported that VSMC phenotypic modulation has an indispensable role in IA pathogenesis, as well as in other vascular disorders [20–22].

Our study also revealed that circ-ATL1 interacts with miR-455. Downregulation of circ-ATL1 was found to promote miR-455 expression, while inhibition of miR-455 was shown to reverse the inhibitory effects of circ-ATL1 silencing on VSMC migration, proliferation and phenotypic modulation. Decreased expression of miR-455 in IA patients has been reported previously [23], while upregulation of miR-455 has been shown to suppress the progression of different cancers [17, 24, 25]. Thus, our findings suggest that downregulation of circ-ATL1 suppressed VSMC migration, proliferation and phenotypic modulation by adsorbing miR-455. Further experiments are required to determine whether other miRNAs, in addition to miR-455, are involved in this process.

We characterized SIRT5 as a downstream target of miR-455 by luciferase reporter analysis. Thus, downregulation of circ-ATL1 inhibits SIRT5 expression, while miR-455 inhibition reverses the inhibitory effects of circ-ATL1 silencing on SIRT5 expression. In addition, overexpression of SIRT5 reversed the inhibitory effects of circ-ATL1 silencing on VSMC proliferation, migration and phenotypic modulation. Previous studies have demonstrated that miR-4735 has an essential role in phenotypic modulation in IA through the regulation of autophagy-dependent VSMC migration and proliferation [26]. Inhibition of autophagy reverses VSMC migration and proliferation. Previously, SIRT5 has been shown to promote autophagy [27]. Thus, our study suggests that downregulation of circ-ATL1 reverses the activation effect of SIRT5 on VSMC proliferation, migration and contractility in IA by adsorbing miR-455, and that these responses may be related to autophagy.

## Conclusion

**Fig. 6:**
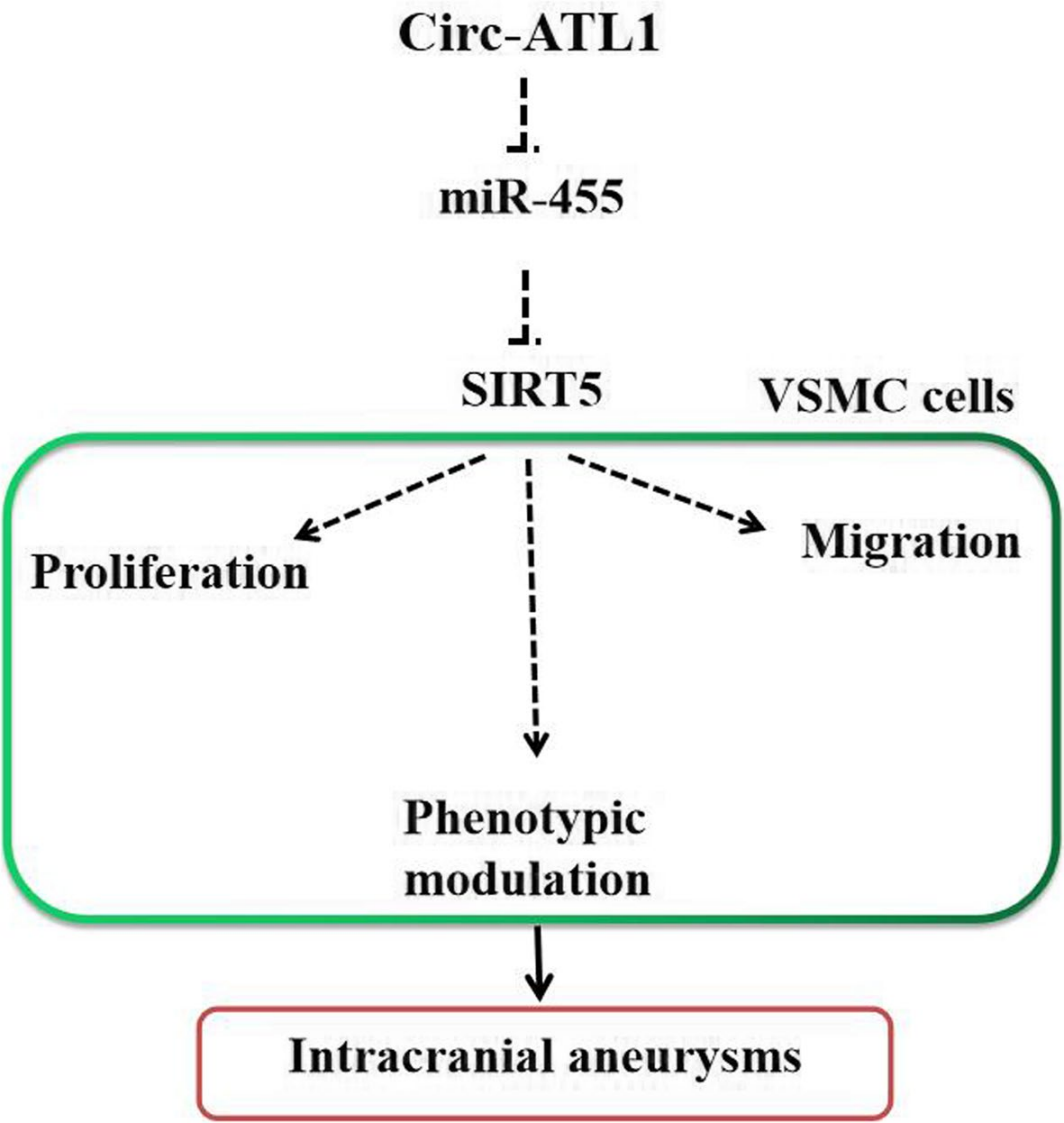
Schematic diagram showing that expression of circ-ATL1 promotes activation of SIRT5 and increases VSMC proliferation, migration and contractility in IA by adsorbing miR-455

Our study found that abnormal expression of circ-ATL1, miR-455 and SIRT5 is correlated with the progression of IA in aneurysm patients. We demonstrated that decreased miR-455 expression or increased circ-ATL1/SIRT5 expression have a role in regulating VSMC function by stimulating VSMCs to switch from a “contractile” phenotype to a “synthetic” phenotype, which correlates to induction of migration and proliferation in vitro. We further showed that SIRT5 overexpression or miR-455 inhibition reverses this switching between phenotypes. Our findings are important for the development of therapeutic targets that can prevent IA progression (Fig. 6).

## Supplementary Information

**Additional file 1.**

## Data Availability

The datasets used and/or analyzed during the current study are available from the corresponding author upon reasonable request.

## Abbreviations

VSMCs: Vascular smooth muscle cells
IAs: Intracranial aneurysms
UIAs: Unruptured intracranial aneurysms
MRI: Magnetic resonance imaging
circRNAs: Circular RNAs
RT: Room temperature
FBS: Fetal bovine serum
si-circ-ATL1: siRNA against circ-ATL1
NC: Negative control
