# Supplementary figures and images for: Circ-ATL1 silencing reverses the activation effects of SIRT5 on smooth muscle cellular proliferation, migration and contractility in intracranial aneurysm by adsorbing miR-455
Source: BMC Mol Cell Biol. 2023 Jan 30;24:3. doi: 10.1186/s12860-022-00461-2 (PMC9887762; doi:10.1186/s12860-022-00461-2)

Fig. 3A

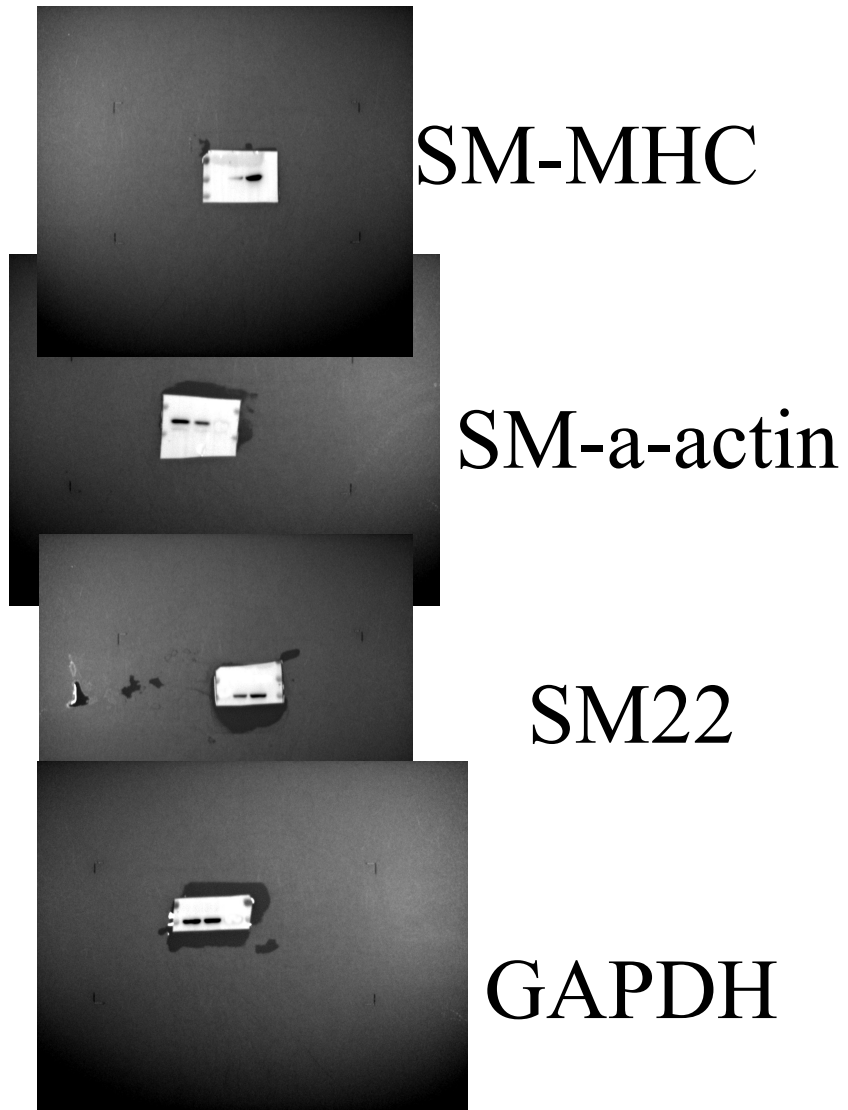

Fig. 3C

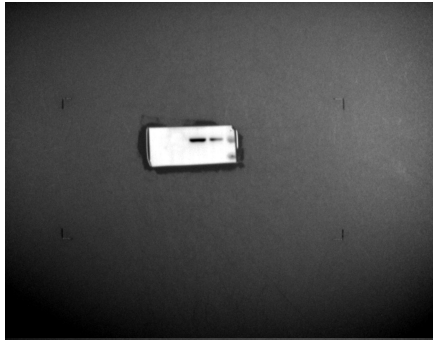

MMP-2

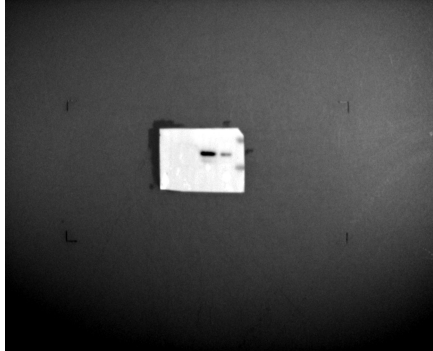

MMP-3

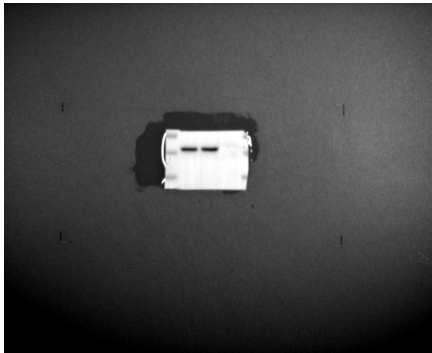

GAPDH

Fig. 5D

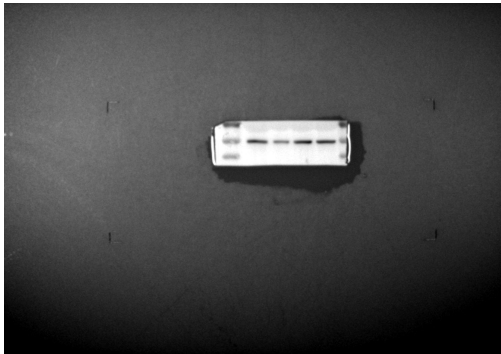

SM-MHC

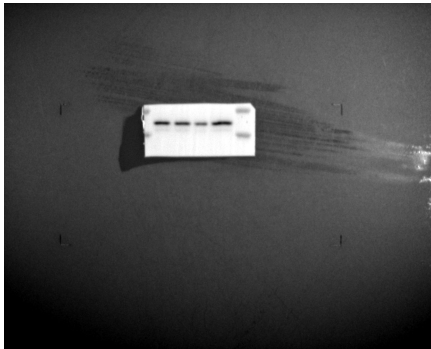

SM-a-actin

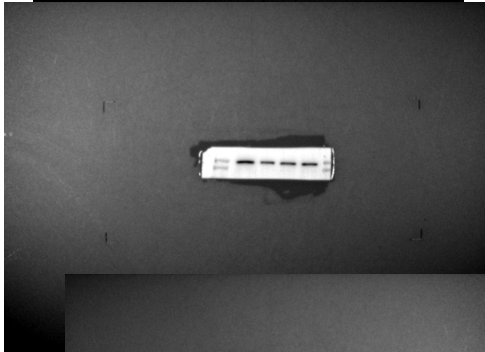

SM22

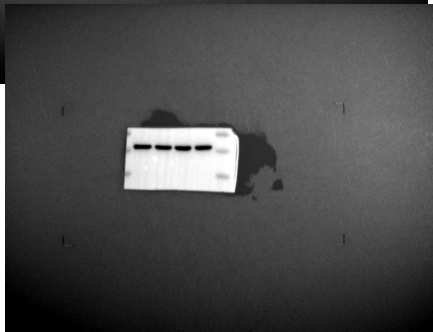

GAPDH

Fig. 5H

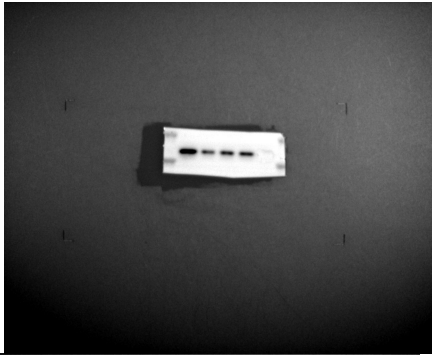

MMP-2

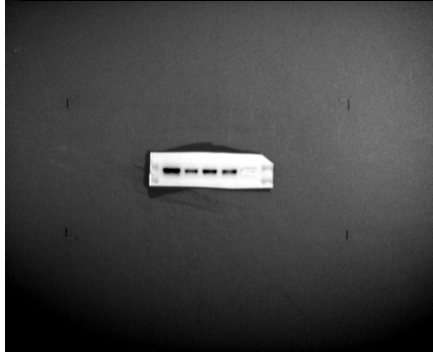

MMP-3

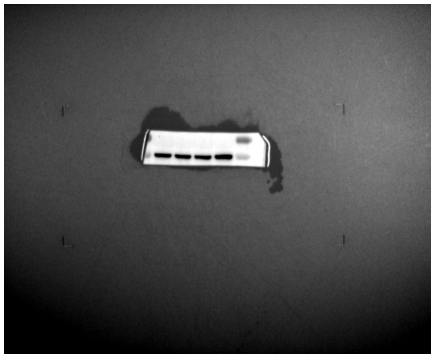

GAPDH

Supplement: Supplementary file 1 — Additional file 1. [file 12860_2022_461_MOESM1_ESM.pdf]
